# Observations on Difficulty of Swallowing

**Published:** 1803-04-01

**Authors:** 


Medical and Phyfical Journal.

VOL. IX.] APRIL 1, 1803. [NO. L.

*Printed for R. PHILLIPS, by W. Thorne, Red Lion Court, Fleet Street, London.*

### OBSERVATIONS *on* DIFFICULTY *of* SWALLOWING; *by* Dr. WICHMANN. *Communicated by Dr. Noehden.*

THE difficulty of swallowing, known by the name of Dysphagia, is undoubtedly a very distressing, and in some cases, a dangerous affection, which deserves the attention of every practitioner, as it is a disease whose diagnosis and particular nature are sometimes misunderstood, and not unfrequently attended with much difficulty. Some species of this affection are easily distinguished, while the nature of other idiopathic affections of the œsophagus requires a more accurate examination for being well understood; a circumstance by which their cure is greatly influenced. Difficult deglutition, considered as an idiopathic affection of the œsophagus, frequently originates in the following causes.

1. Diminished capacity of the œsophagus.
2. Spasms of the œsophagus.
3. Paralytic state of the organ, and relaxation of its membranes.

The general decursus morbi is so extremely similar in these different species, that they are easily taken for each other. The organs of deglutition are affected, the deglution is obstructed, which gradually increases, till not any thing is possible to be got into the stomach. Sometimes the disease proceeds so slowly, as not even to be perceived by the patient till after its having arrived at a certain degree of danger.

Those species of the malady are characterized by the kind of vomition with which they are accompanied, and which may rather be called a rumination, as it is merely produced by the muscles of the œsophagus, without the abdominal muscles taking any share in it. It is not attended either with nausea or any other disagreeable sensation, which constitutes the forerunner of common vomition. The appetite remains good. There are cases in which this rumination is observed for a long time, without any other symptom; the patient brings up the ingesta without great exertion; they get down again in small portions, and nothing remains in the mouth which ought to be thrown out. Thus far the abovementioned species of Dysphagia agree with one another; but they differ in other respects, which must be carefully attended to, in order to be able to distinguish them.

It is not uncommon that other membranes get thicker, and indurated; a circumstance by which their function is greatly disturbed, and several morbid phenomena produced. This also sometimes takes place in the œsophagus, the internal membrane of which grows thick and indurated, which obstructs the action of the muscular fibres and the deglutition, by diminishing the capacity or space of the œsophagus.

A constriction of the œsophagus likewise arises from indurations or scirrhosities at any single spot of that organ; from a pressure on it by an indurated gland, &c.; but all which causes are not the subject of our inquiry. A diminished capacity of the œsophagus may be assigned as the most common cause of Dysphagia. The patient feels a little pain, pressure, or tension down the œsophagus, or up to the shoulder blade; he fancies that he feels at a certain place as it were, a stoppage, and he clearly discovers the impediment in the œsophagus. The more downwards this seems to be, the more certainly we may conclude on an induration of the œsophagus; and if the disagreeable sensation extends itself to the cardia, it denotes a constriction, induration, and even a cartilaginous scirrhus of this part. If the patient feels the impediment in the 4th or 5th vertebra dorsi, it is very probable that the neighbouring glands are indurated. In this case, the sensation of pain and tension continues even after the deglutition, which is besides, more easy whenever the patient lies on his back. In this species of the disease, the ingesta are brought up again four or five hours after the meal; at first in a small quantity, but as the malady advances, a greater portion comes up within a shorter time after they have been taken.

The second species of Dysphagia, originating in a spasm of the œsophagus, is distinguished by the following characteristics. 1. The disease seems to make a remission towards the evening, when the patient swallows with less difficulty; difficulty. This peculiarity, however, is only to be remarked at the beginning of the disease. — 2. The impediment of deglutition is generally felt in the inferior part of the œsophagus, near the stomach, from whence the disagreeable sensation extends to the stomach itself. — 3 The difficulty of swallowing comes on suddenly, and is immediately attended with a painful sensation. — 4. Warm liquors get easier down the stomach than cold ones. — 5. Liquors, slowly swallowed, go down to the stomach without any difficulty; whereas, if drank at once, or in large portions, they are immediately thrown up. — 6. Other parts of the body are found to be equally affected by spasms.

The third species of Dysphagia is distinguished by the following phenomena. 1. It, for the most part, occurs in old people, being frequently produced by apoplectic fits. — 2. The patient gets down solid food much easier than liquors. — 3. The patient cannot point out the certain place where he feels the impediment. — 4. No painful sensation, tension, or pressure is to be perceived in this case. — 5. We can trace no impediment by means of a probe. Sometimes it is very difficult to distinguish this species of Dysphagia from that we have first mentioned.

There is another species of this complaint, nearly related to the latter, namely, that which arises from a relaxation of the membranes of the pharynx and from folds in the œsophagus. This affection, which must be deemed incurable, occurs very rarely, and particularly takes place in the upper part of the œsophagus and the pharynx, where it forms a pharyngocele. While the patient makes an attempt to swallow, he presses the ingesta into the sac, by which the œsophagus is entirely blocked up. These folds are very small at the beginning, increasing by degrees. Some remainders of food are then brought up by a sort of reaching, and a particular hissing sound is often heard. The vomition ensues soon after the taking of the food, and the patient throws it out, mixed with much mucus, which had been collected in the sac. This dangerous complaint sometimes supervenes to other species of Dysphagia, as, from the frequent attempts to swallow, the internal membrane begins to slacken; it is, however, to be particularly met with in old people.

In the first species of Dysphagia, mercurials are found of great service; but they are of no effect, indeed rather prejudicial, in other species of this disease. Of all the preparations of quicksilver, the corrosive sublimate seems to be particularly efficacious in this case. The spasmodic

Dysphagia indicates the use of camphor, opium, musk, &c. and in a paralytic case, large doses of extract. quassiæ were very serviceable. If the disease originates from a relaxation of the membranes of the pharynx, we should abstain from tormenting the unhappy patient with any kind of medicines, however distressing his state may be. The only thing to be done is to apply nourishing clysters.

